# Supplementary material for: Ultrasensitive and visual detection of SARS-CoV-2 using all-in-one dual CRISPR-Cas12a assay
Source: Nat Commun. 2020 Sep 18;11:4711. doi: 10.1038/s41467-020-18575-6 (PMC7501862; doi:10.1038/s41467-020-18575-6)
Supplement: Supplementary file 1 — Supplementary Information [file 41467_2020_18575_MOESM1_ESM.pdf]

Supplementary information

Ultrasensitive and visual detection of SARS-CoV-2 using all-in-one dual CRISPR-Cas12a (AIOD-CRISPR) assay

Xiong Ding et al.

Supplementary Figures 1-16

Supplementary Tables 1 and 2

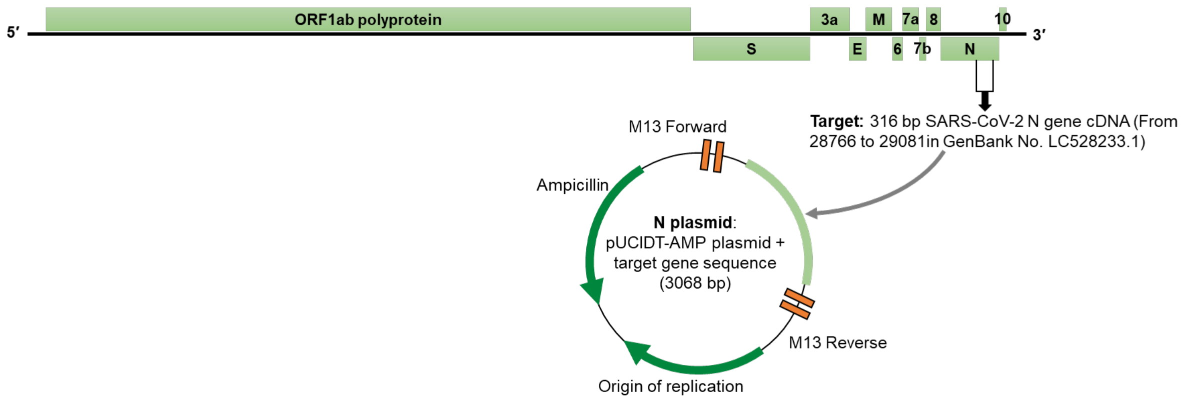

Supplementary Fig. 1. The pUCIDT-AMP plasmid (from IDT) containing the 316 bp SARS-CoV-2 N gene cDNA (N plasmid) fragment and its location at the genome map.

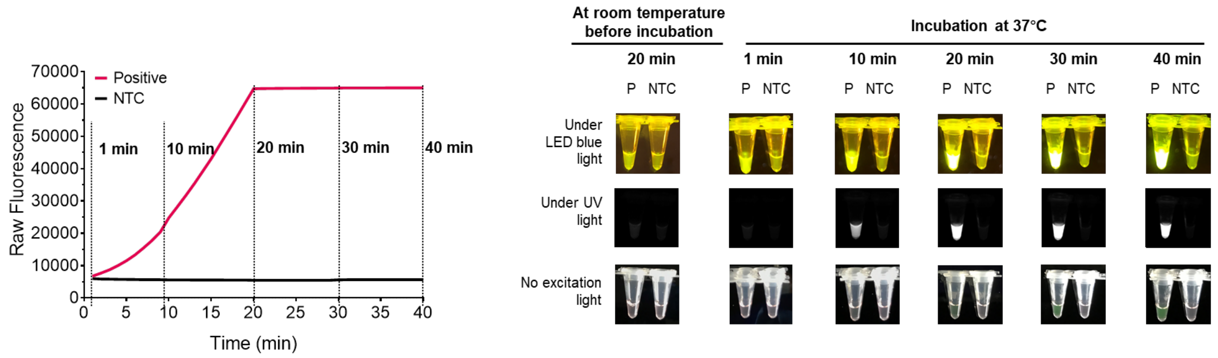

Supplementary Fig. 2. The AIOD-CRISPR assay incubated at room temperature for 20 min and at 37°C for 1, 10, 20, 30, and 40 min. Positive (P), 3× 10^3 copies of the plasmid DNA containing SARS-CoV-2 N gene sequence. NTC, non-target control reaction.

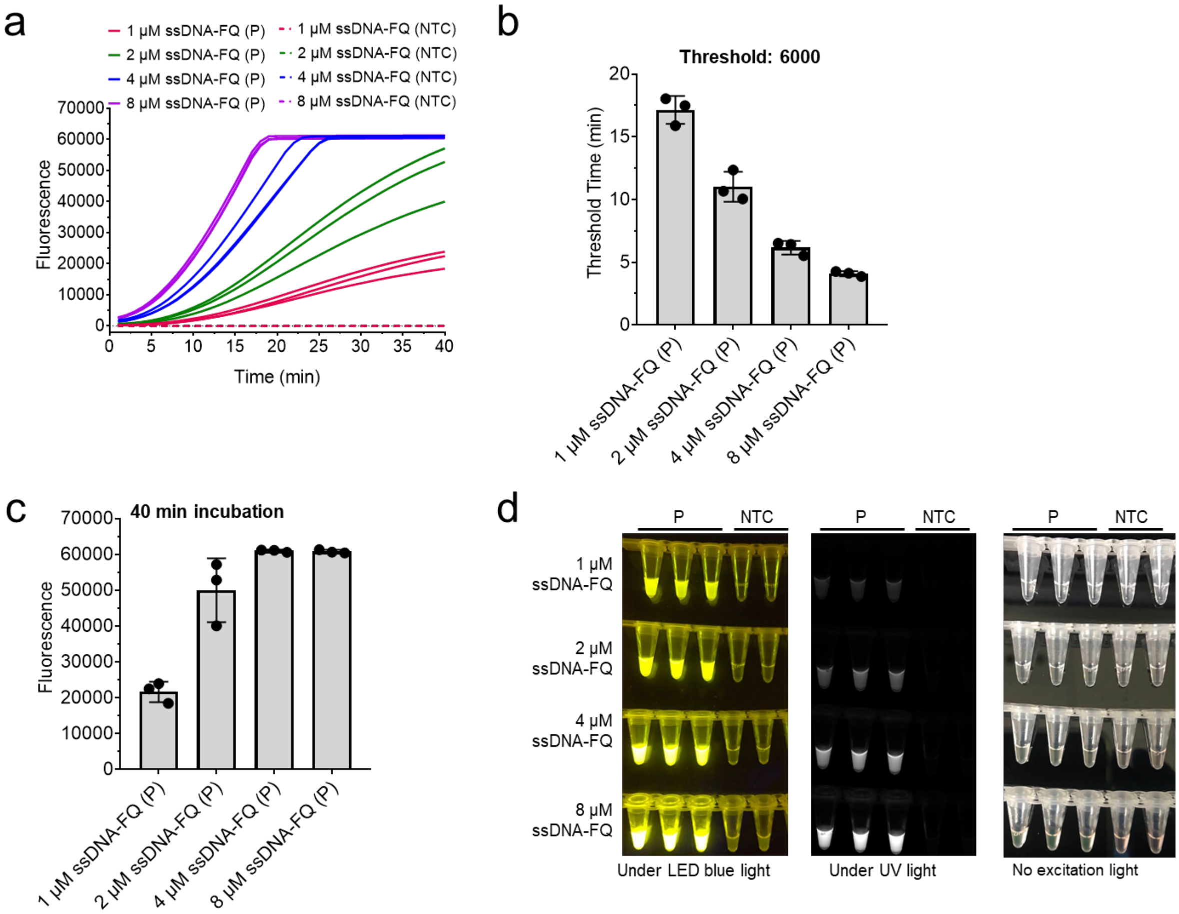

Supplementary Fig. 3. The AIOD-CRISPR assay with various concentrations of the ssDNA-FQ reporters. **a** Real-time fluorescence detection. **b** Threshold time comparison. Source data are provided as a Source Data file. **c** Endpoint fluorescence intensity comparison after 40 min incubation. Source data are provided as a Source Data file. **d** Visual detection comparison after 40 min incubation. P, 3× 10^3 copies of the plasmid DNA containing SARS-CoV-2 N gene sequence. NTC, non-target control reaction. Error bars represent the means ± s.d. from three replicates (n = 3).

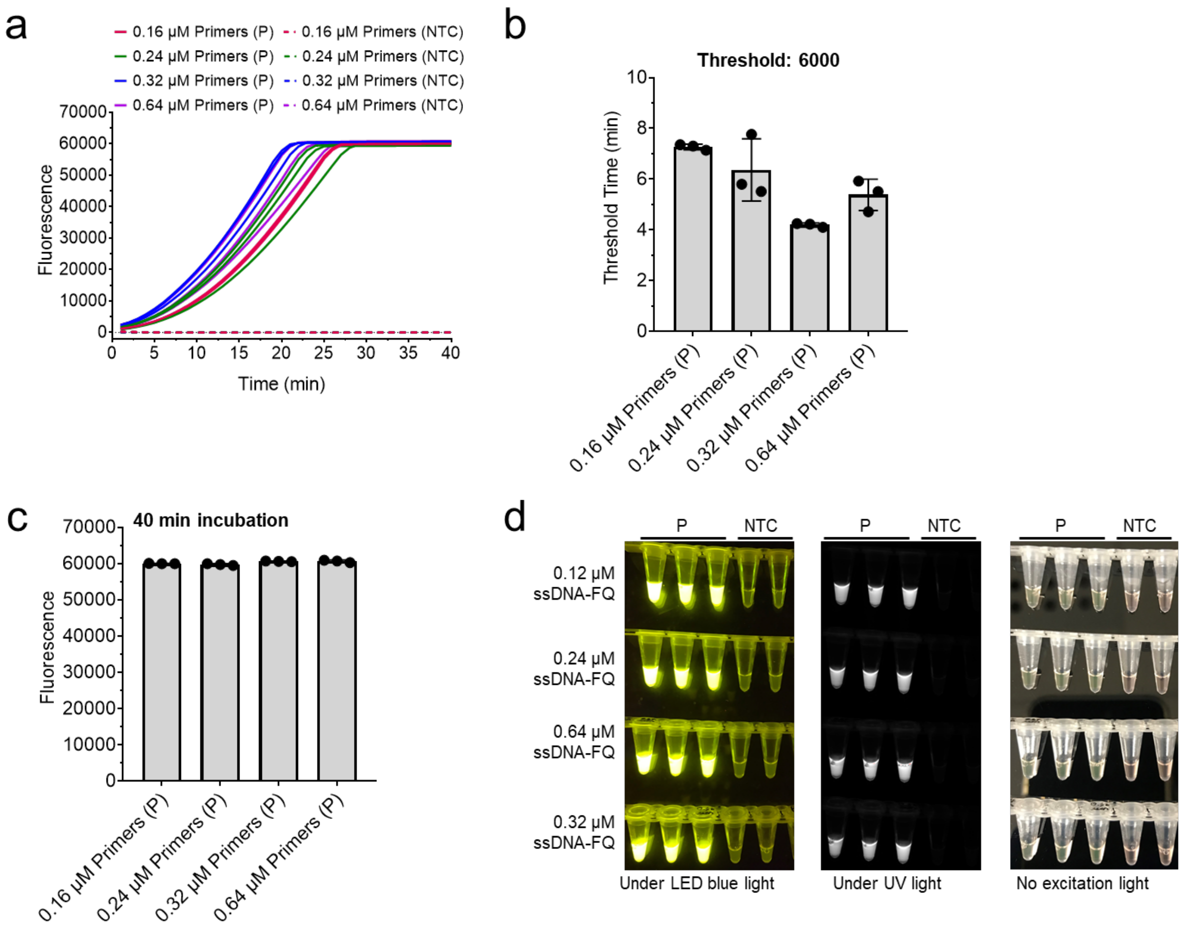

Supplementary Fig. 4. The AIOD-CRISPR assay with various concentrations of the primers. **a** Real-time fluorescence detection. **b** Threshold time comparison. Source data are provided as a Source Data file. **c** Endpoint fluorescence intensity comparison after 40 min incubation. Source data are provided as a Source Data file. **d** Visual detection comparison after 40 min incubation. P, 3× 10^3 copies of the plasmid DNA containing SARS-CoV-2 N gene sequence. NTC, non-target control reaction. Error bars represent the means ± s.d. from three replicates (n = 3).

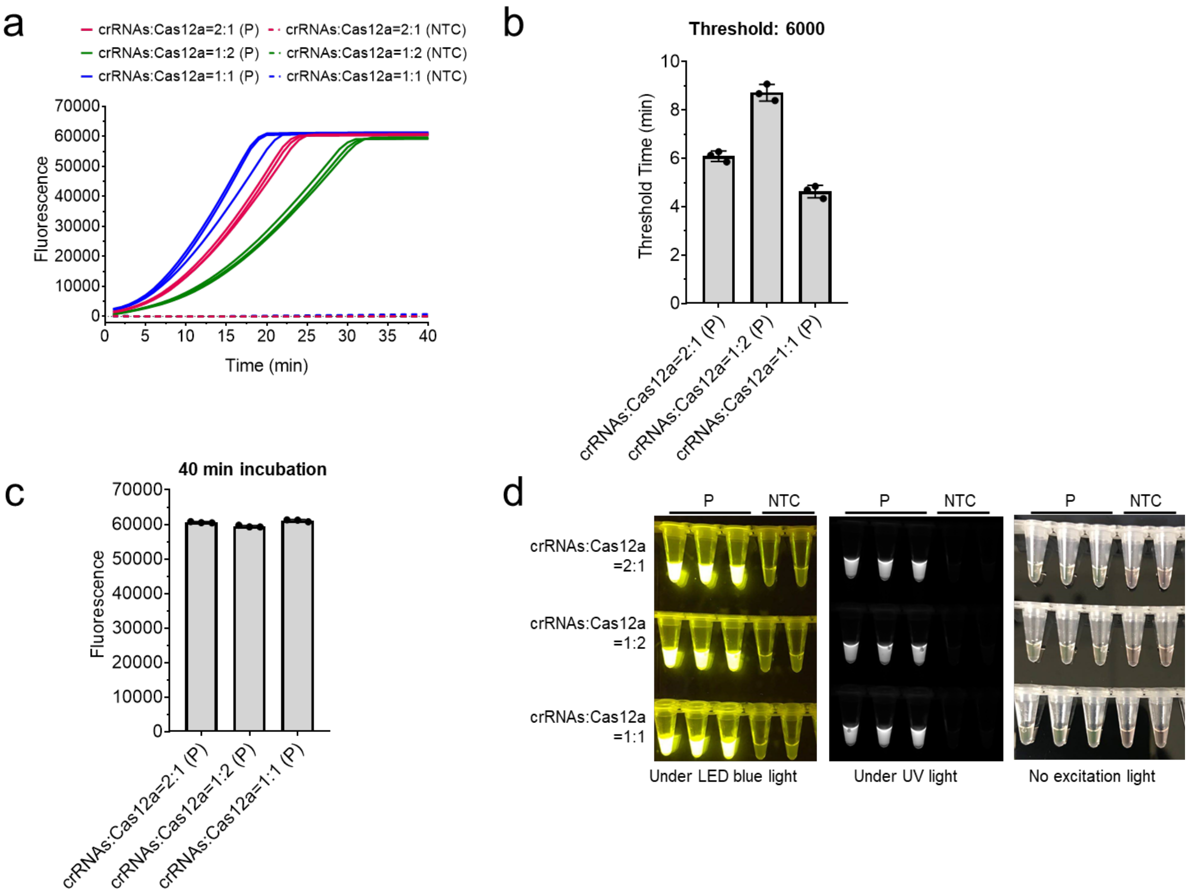

Supplementary Fig. 5. The AIOD-CRISPR assay with various ratios of crRNAs to Cas12a (the concentration of each of crRNAs was fixed at 0.64 μM). **a** Real-time fluorescence detection. **b** Threshold time comparison. Source data are provided as a Source Data file. **c** Endpoint fluorescence intensity comparison after 40 min incubation. Source data are provided as a Source Data file. **d** Visual detection comparison after 40 min incubation. P, 3× 10^3 copies of the plasmid DNA containing SARS-CoV-2 N gene sequence. NTC, non-target control reaction. Error bars represent the means ± s.d. from three replicates (n = 3).

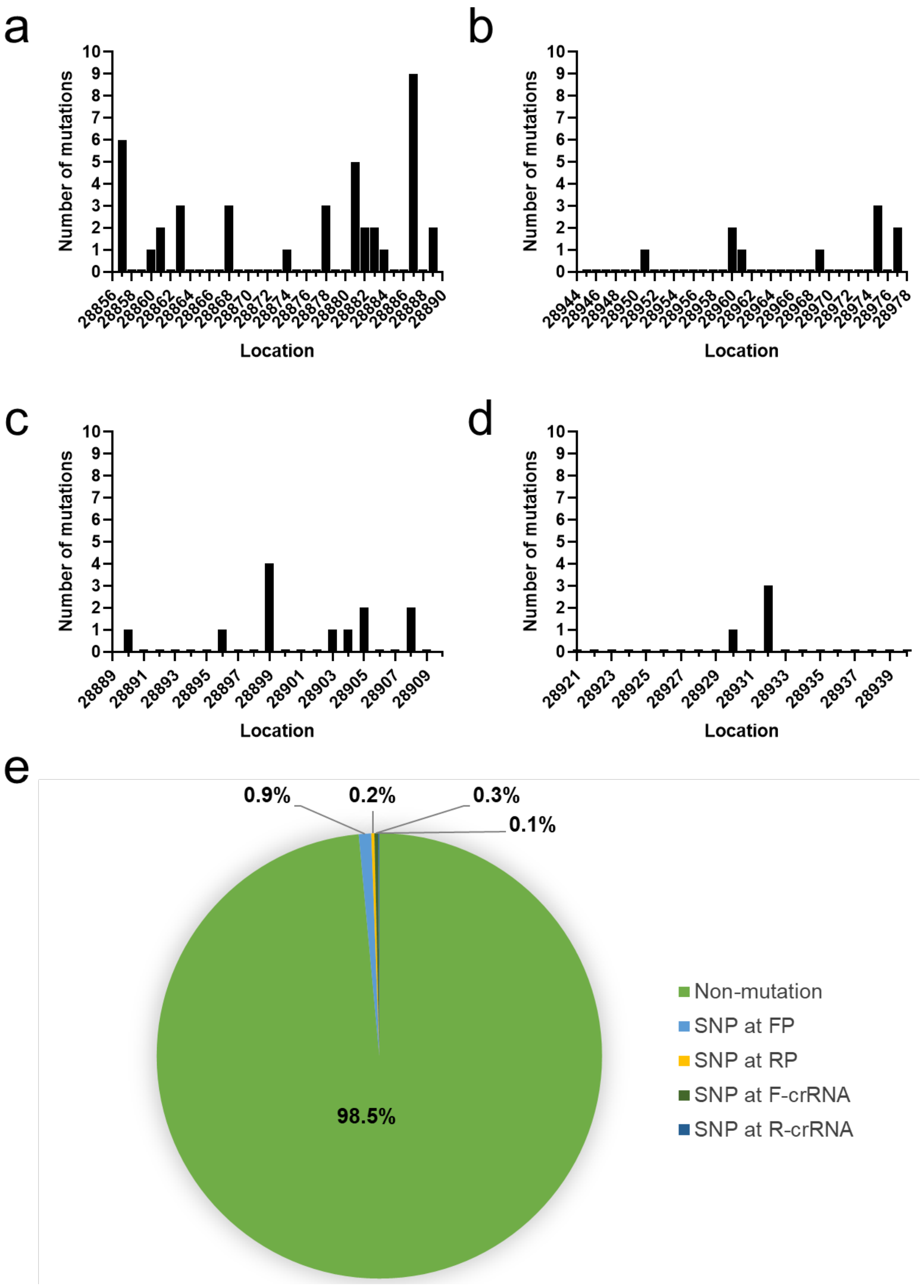

Supplementary Fig. 6. Number of mutations at each nucleotide location in the primers and crRNAs of AIOD-CRISPR assays based on multiple sequence alignments analysis of 4463 SARS-CoV-2 genomes sampled between December 2019 and August 2020. **a-d** Number of mutations at each nucleotide location in forward primer (FP), reverse primer (FP), forward crRNA (F-crRNA), and reverse crRNA (R-crRNA). **e** Percentage of the single nucleotide polymorphism (SNP) mutation when considering each mutation as a SNP mutation. The data were from GISAID-provided genomic epidemiology of hCoV-19 (as of August 14, 2020) (https://www.gisaid.org/epiflu-applications/next-hcov-19-app/). Source data are provided as a Source Data file.

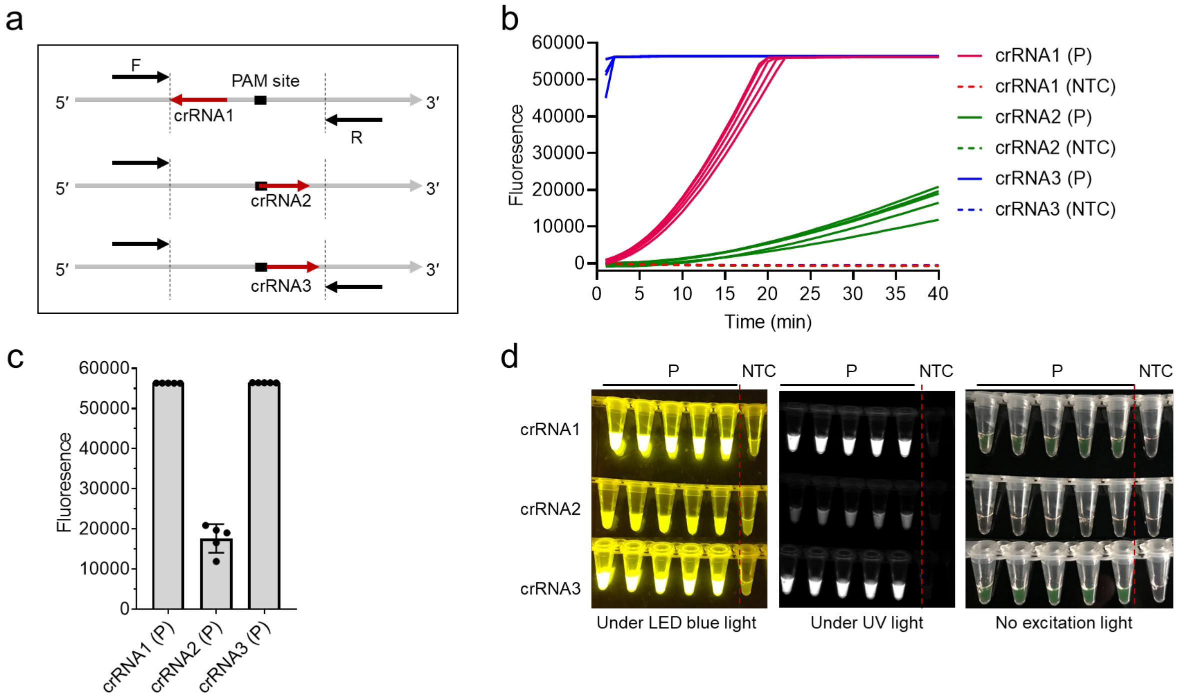

Supplementary Fig. 7. Comparison of AIOD-CRISPR assays using different single crRNA for the detection of 3×10^{6} copies of plasmid DNA containing SARS-CoV-2 N gene sequence (N plasmid). **a** Primer design and the location of each crRNA. Among them, the design of crRNA1 and crRNA2 were not limited by the PAM site, while the design of crRNA3 was limited by the PAM site. Detailed sequences were displayed in Fig. 2a. **b** Real-time fluorescence monitoring curves. **c** Endpoint fluorescence comparison after 40 min incubation. Source data are provided as a Source Data file. **d** Visual detection comparison after 40 min incubation. Five replicates were run (n=5) for each positive reaction. P, the positive reaction with 3×10^{6} copies N plasmids. NTC, non-template control reaction. Error bars represent the means ± s.d. from replicates.

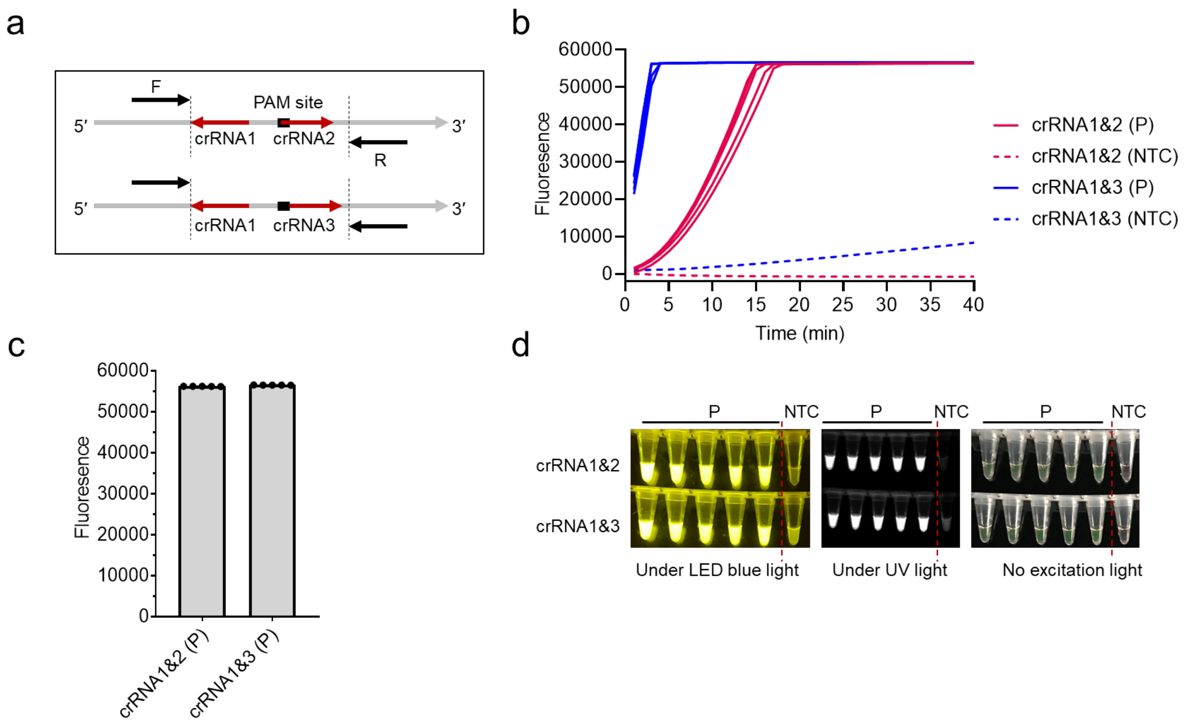

Supplementary Fig. 8. Comparison of AIOD-CRISPR assays using different pairs of dual crRNAs for the detection of 3×10^6 copies of plasmid DNA containing SARS-CoV-2 N gene sequence (N plasmid). **a** Design and location of primers and crRNAs. Detailed sequences were displayed in Fig. 2a. **b** Real-time fluorescence monitoring curves. **c** Endpoint fluorescence comparison after 40 min incubation. Source data are provided as a Source Data file. **d** Visual detection comparison after 40 min incubation. Five replicates were run for each positive reaction (n=5). P, the positive reaction with 3×10^6 copies N plasmids. NTC, non-template control reaction. Error bars represent the means ± s.d. from replicates.

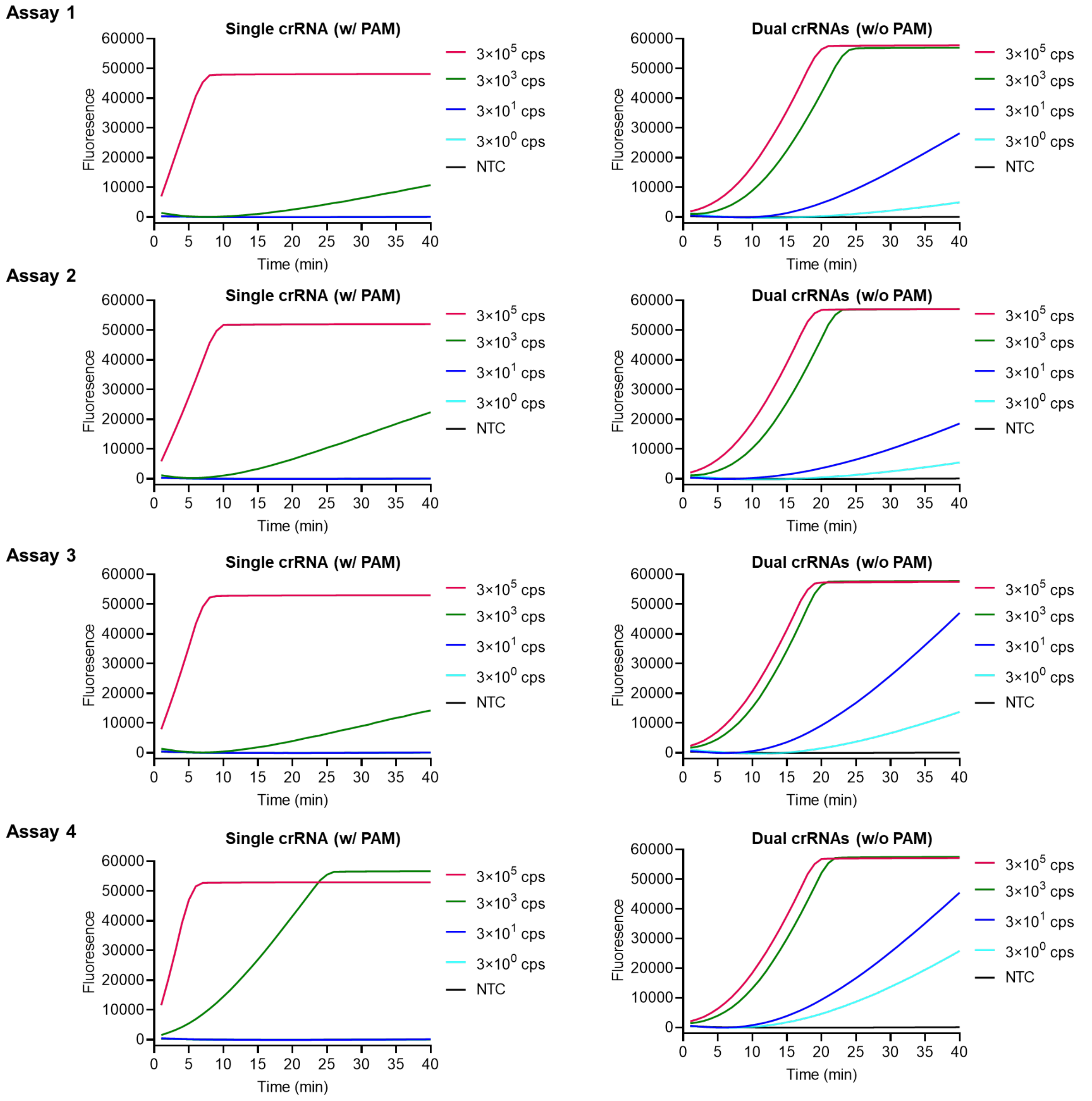

Supplementary Fig. 9. Sensitivity comparison of AIOD-CRISPR assays using single crRNA (crRNA3) with PAM site limitation or the dual crRNAs (crRNA1&2) without PAM site limitation for detection of the ten-fold serial dilution of plasmid DNA containing SARS-CoV-2 N gene sequence. Detailed sequences were displayed in Fig. 2a. Four independent assays were conducted. NTC, non-template control reaction.

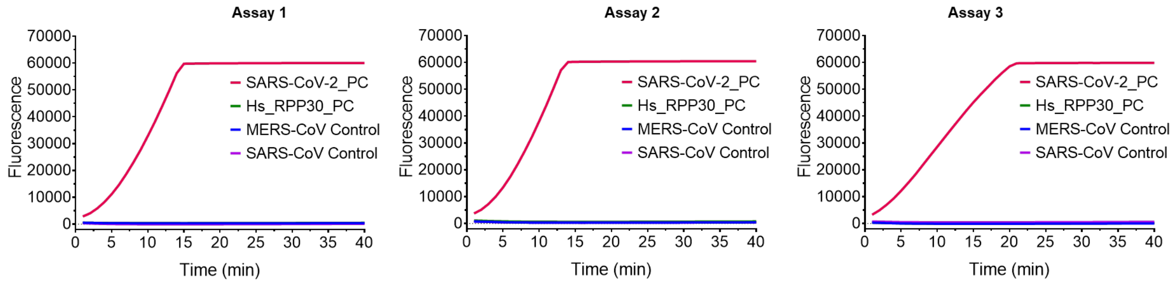

Supplementary Fig. 10. AIOD-CRISPR specificity for SARS-CoV-2 N detection in three independent assays. IDT company provided the complete N gene from SARS-CoV-2 (SARS-CoV-2 _PC, Catalogue # 10006625, IDT), SARS (SARS-CoV_PC, Catalogue # 10006624, IDT), and Middle East respiratory syndrome (MERS) (MERS-CoV (Middle East respiratory syndrome coronavirus)_PC, Catalogue # 10006623, IDT), as well as the Hs_RPP30 control (Hs_RPP30_PC, Catalogue # 10006626, IDT) with a portion of human RPP30 gene.

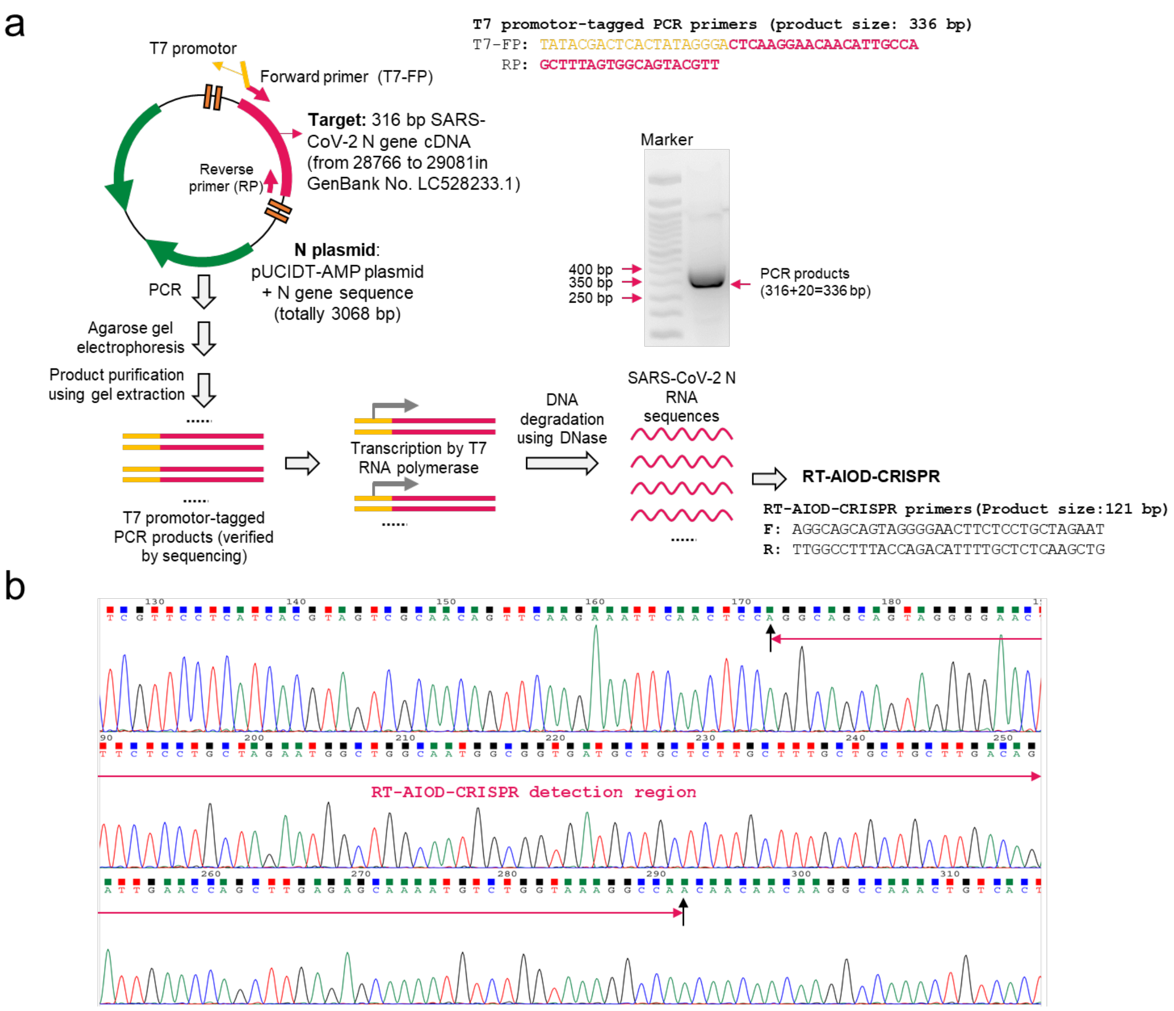

Supplementary Fig. 11. a Protocol and PCR primers for preparing the SARS-CoV-2 N RNA sequences. **b** Sanger sequencing of the RT-AIOD-CRISPR detection region in the prepared SARS-CoV-2 N RNA.

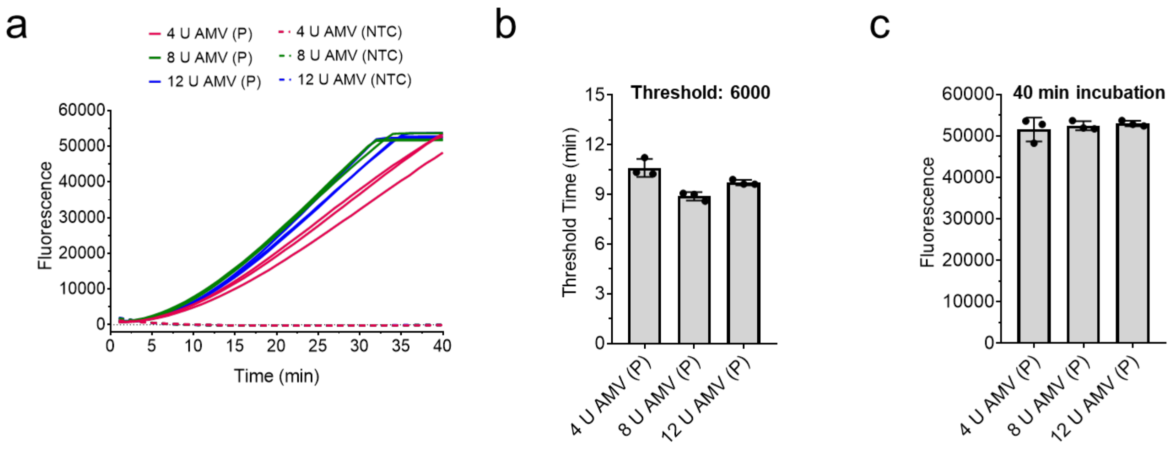

Supplementary Fig. 12. The RT-AIOD-CRISPR assay using various concentrations of AMV reverse transcriptase. **a** Real-time fluorescence detection. **b** Threshold time comparison. Source data are provided as a Source Data file. **c** Endpoint fluorescence intensity comparison after 40 min incubation. Source data are provided as a Source Data file. P, the positive reaction with 5× 10^3 copies of SARS-CoV-2 N RNA sequences. NTC, non-target control reaction. Three replicates were run for each reaction or test. Error bars represent the means ± s.d. from replicates.

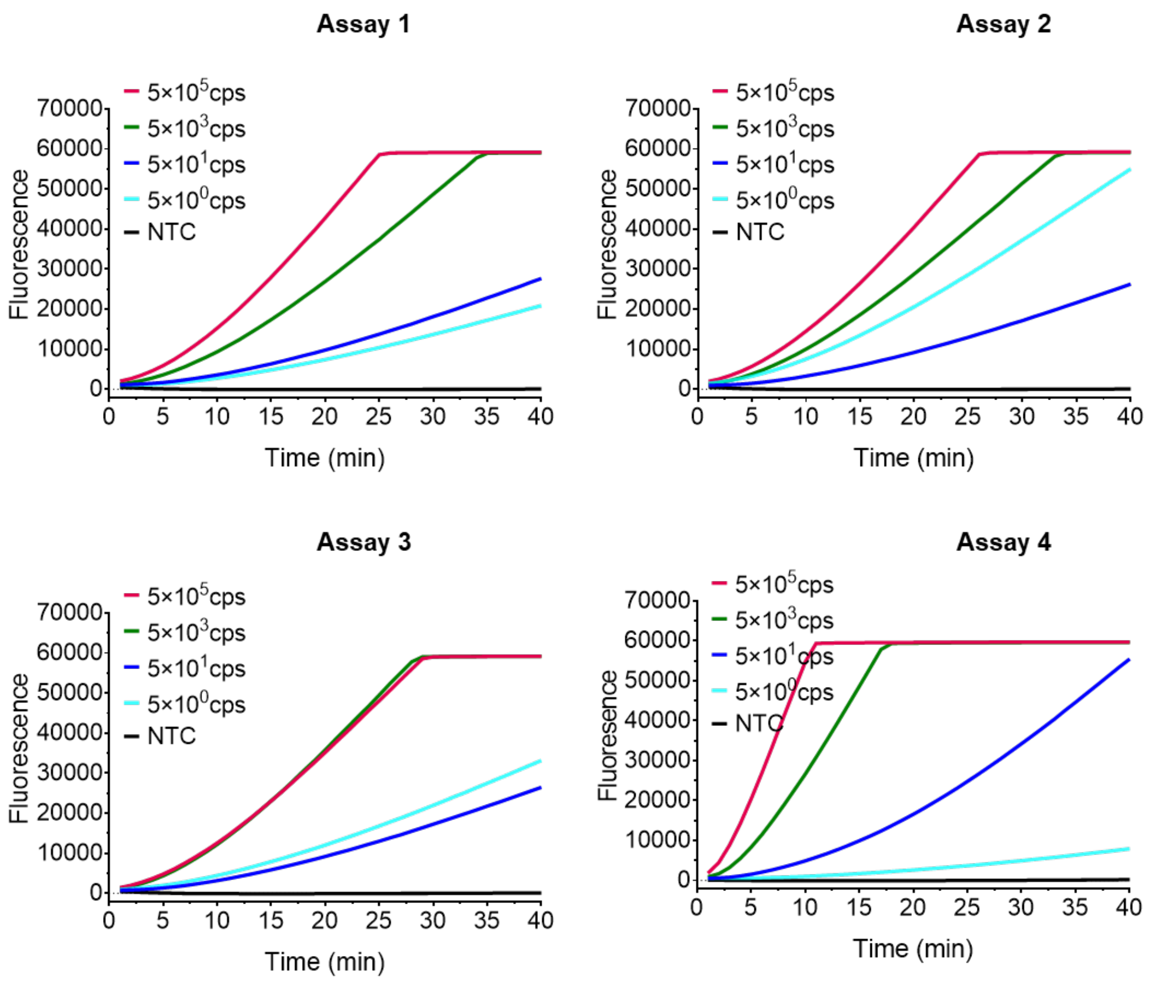

Supplementary Fig. 13. Sensitivity of RT-AIOD-CRISPR assay for the detection of SARS-CoV-2 synthetic RNA templates in four independent assays. NTC, non-target control reaction.

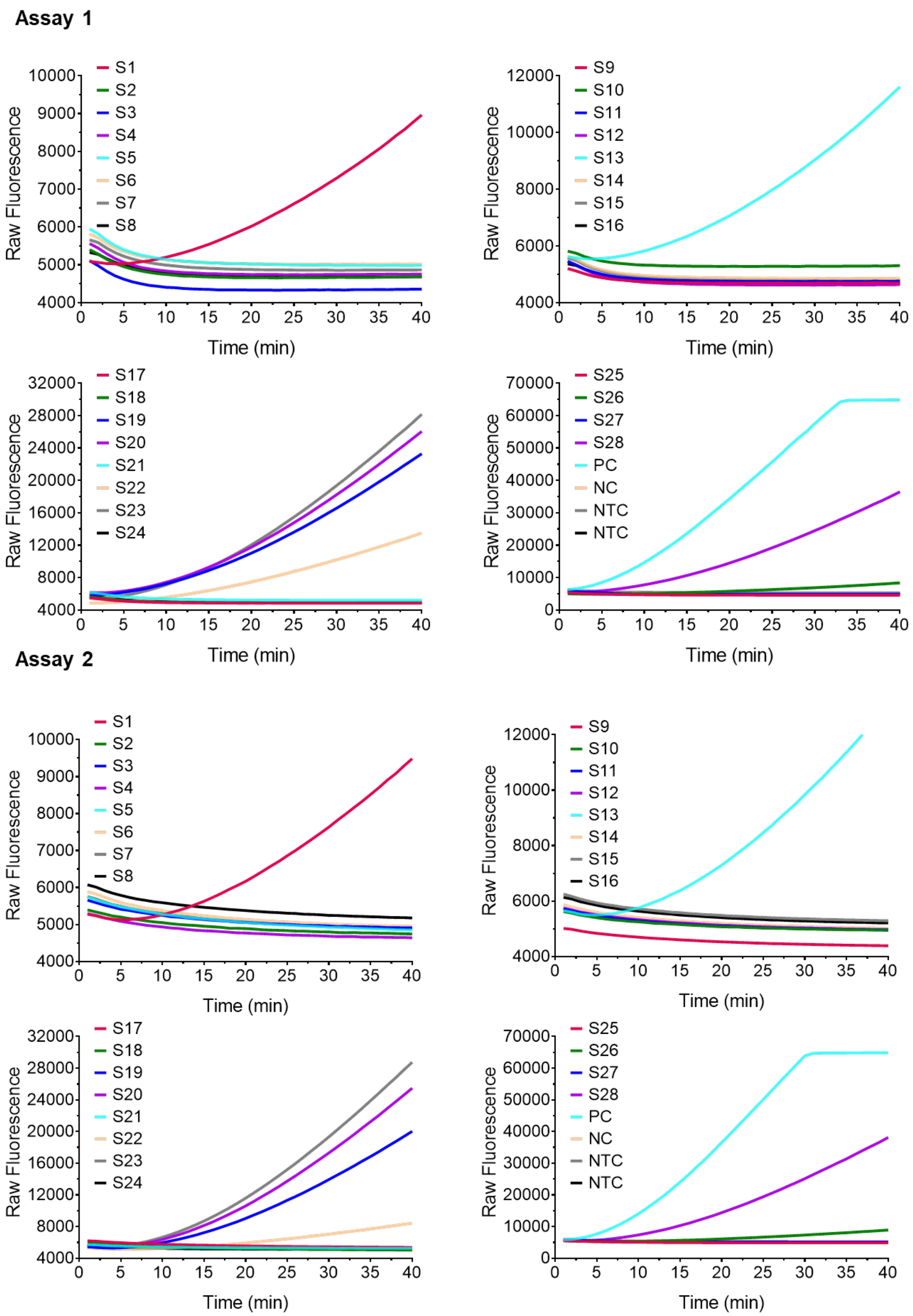

Supplementary Fig. 14. Real-time RT-AIOD-CRISPR assay for SARS-CoV-2 detection in 28 clinical swab samples in two independent assays. Positive control (PC), 1.2×10^{6} of synthetic SARS-CoV-2 N RNA. S1-S28, clinical sample 1-28. NC, SARS-CoV-2-negative control. NTC, non-template control reaction.

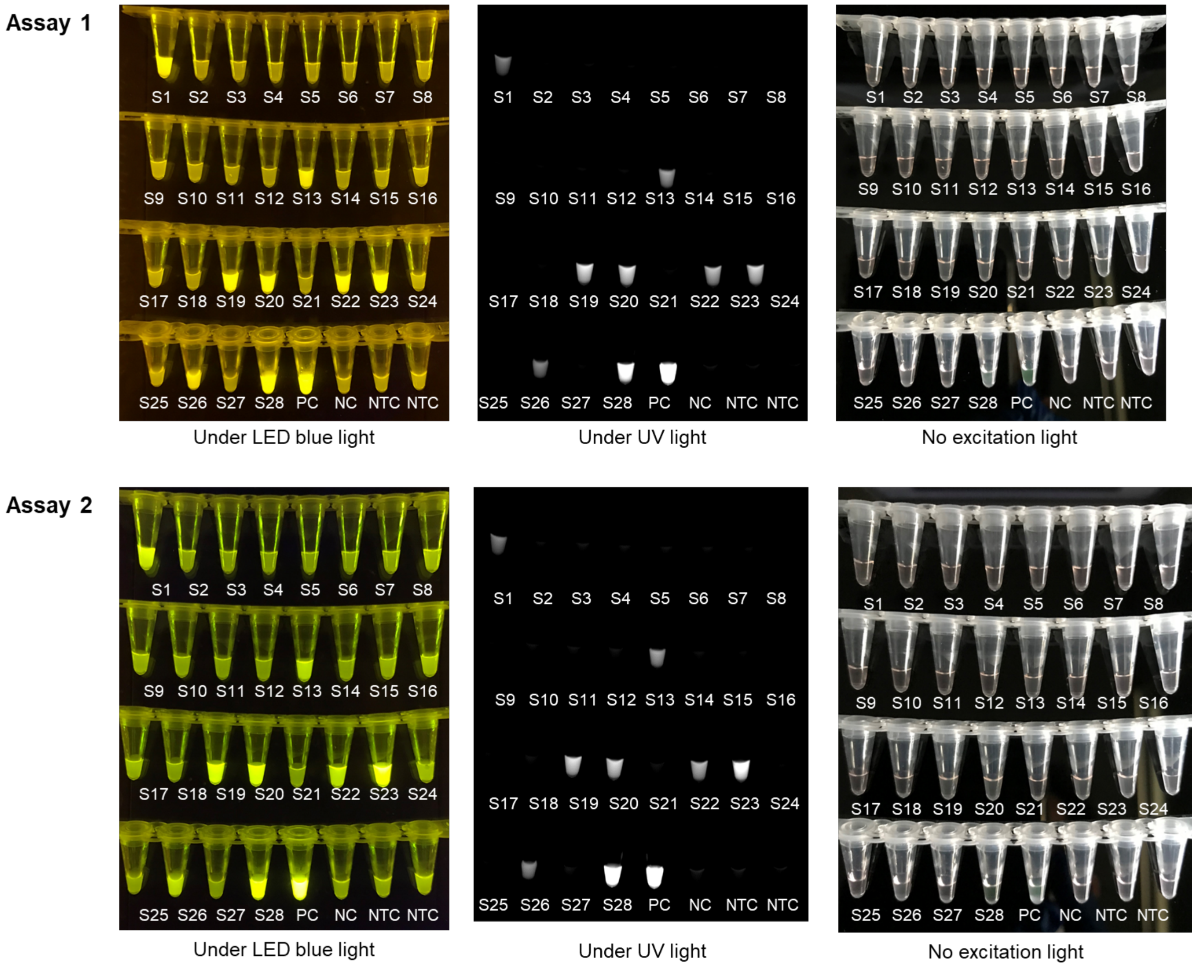

Supplementary Fig. 15. Endpoint fluorescence/visual RT-AIOD-CRISPR assay for SARS-CoV-2 detection in 28 clinical swab samples in two independent assays. Positive control (PC), 1.2×10^6 of synthetic SARS-CoV-2 N RNA. S1-S28, clinical sample 1-28. NC, SARS-CoV-2-negative control. NTC, non-template control reaction.

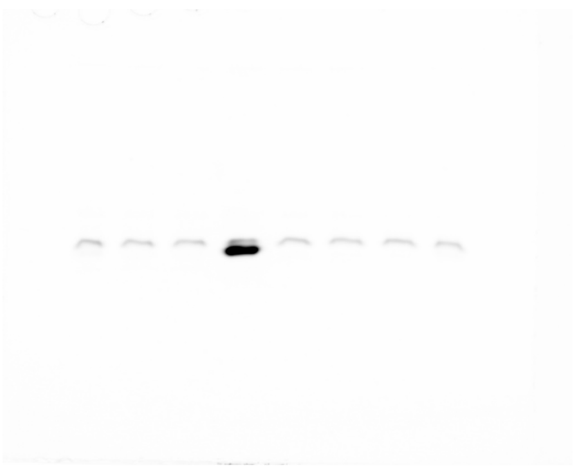

Supplementary Fig. 16. Uncropped PAGE image of Fig. 1b.

Supplementary Table 1. Comparison of RT-AIOD-CRISPR and CDC-approved RT-PCR assay for detection of SARS-CoV-2 in 28 clinical swab samples

No.	Real-time RT-AIOD-CRISPR	Visual RT-AIOD-CRISPR	CDC-proved RT-qPCR
Sample 1	+ + [a]	+ + [a]	+ [b]
Sample 2	- -	- -	-
Sample 3	- -	- -	-
Sample 4	- -	- -	-
Sample 5	- -	- -	-
Sample 6	- -	- -	-
Sample 7	- -	- -	-
Sample 8	- -	- -	-
Sample 9	- -	- -	-
Sample 10	- -	- -	-
Sample 11	- -	- -	-
Sample 12	- -	- -	-
Sample 13	+ +	+ +	+
Sample 14	- -	- -	-
Sample 15	- -	- -	-
Sample 16	- -	- -	-
Sample 17	- -	- -	-
Sample 18	- -	- -	-
Sample 19	+ +	+ +	+
Sample 20	+ +	+ +	+
Sample 21	- -	- -	-
Sample 22	+ +	+ +	+
Sample 23	+ +	+ +	+
Sample 24	- -	- -	-
Sample 25	- -	- -	-
Sample 26	+ +	+ +	+
Sample 27	- -	- -	-
Sample 28	+ +	+ +	+

[a] SARS-CoV-2 was detected twice by RT-AIOD-CRISPR assay in two independent assays.
[b] SARS-CoV-2 was detected once by CDC-approved RT-PCR assay (Thermo Fisher Scientific Inc., Waltham, MA) prior to our AIOD-CRISPR assay

Supplementary Table 2. The sequence list of primers, crRNAs and target inserted into a plasmid

Item	Sequence (5'-3')
The 316 bp SARS-CoV-2 N gene sequence inserted into the pUCIDT (Amp) plasmid	CTCAAGGAACAACATTGCCAAAAGGCTTCTACGCAGAAGGGAGCAGAGGCGGCAGTCAAGCCTCTTCTCGTTCCTCATCACGTAGTCGCAACAGTTCAAGAAATTCAACTCCAGGCAGCAGTAGGGGAACTTCTCCTGCTAGAATGGCTGGCAATGGCGGTGATGCTGCTCTTGCTTTGCTGCTGCTTGACAGATTGAACCAGCTTGAGAGCAAAATGTCTGGTAAAGGCCAACAACAACAAGGCCAAACTGTCACTAAGAAATCTGCTGCTGAGGCTTCTAAGAAGCCTCGGCAAAAACGTACTGCCACTAAAGC
Forward primer (FP) targeting SARS-CoV-2 N gene	AGGCAGCAGTAGGGGAACTTCTCCTGCTAGAAT
Reverse primer (FP) targeting SARS-CoV-2 N gene	TTGGCCTTTACCAGACATTTTGCTCTCAAGCTG
Forward crRNA (crRNA1) targeting SARS-CoV-2 N gene	UAAUUUCUACUAAGUGUAGAUCAUCACCGCCAUUGCCAGCC
Reverse crRNA (crRNA2) targeting SARS-CoV-2 N gene	UAAUUUCUACUAAGUGUAGAUUUGCUGCUGCUUGACAGAUU
Reverse crRNA (crRNA3) targeting SARS-CoV-2 N gene	UAAUUUCUACUAAGUGUAGAUCUGCUGCUUGACAGAUUGAAC
T7 promotor-tagged PCR forward primer targeting SARS-CoV-2 N gene	TATACGACTCACTATAGGGACTCAAGGAACAACATTGCCA
PCR reverse primer targeting SARS-CoV-2 N gene	GCTTTAGTGGCAGTACGTT
