# Supplementary material for: Ultrasensitive and visual detection of SARS-CoV-2 using all-in-one dual CRISPR-Cas12a assay
Source: Nat Commun. 2020 Sep 18;11:4711. doi: 10.1038/s41467-020-18575-6 (PMC7501862; doi:10.1038/s41467-020-18575-6)
Supplement: Supplementary file 3 — Reporting Summary [file 41467_2020_18575_MOESM3_ESM.pdf]

nature research

Corresponding author(s): Changchun Liu

Last updated by author(s): Aug 18, 2020

Reporting Summary

Nature Research wishes to improve the reproducibility of the work that we publish. This form provides structure for consistency and transparency in reporting. For further information on Nature Research policies, see our Editorial Policies and the Editorial Policy Checklist.

Statistics

For all statistical analyses, confirm that the following items are present in the figure legend, table legend, main text, or Methods section.

n/a	Confirmed	
☐	☒	The exact sample size (n) for each experimental group/condition, given as a discrete number and unit of measurement
☐	☒	A statement on whether measurements were taken from distinct samples or whether the same sample was measured repeatedly
☐	☒	The statistical test(s) used AND whether they are one- or two-sided<br>*Only common tests should be described solely by name; describe more complex techniques in the Methods section.*
☒	☐	A description of all covariates tested
☒	☐	A description of any assumptions or corrections, such as tests of normality and adjustment for multiple comparisons
☐	☒	A full description of the statistical parameters including central tendency (e.g. means) or other basic estimates (e.g. regression coefficient) AND variation (e.g. standard deviation) or associated estimates of uncertainty (e.g. confidence intervals)
☐	☒	For null hypothesis testing, the test statistic (e.g. F, t, r) with confidence intervals, effect sizes, degrees of freedom and P value noted<br>*Give P values as exact values whenever suitable.*
☒	☐	For Bayesian analysis, information on the choice of priors and Markov chain Monte Carlo settings
☒	☐	For hierarchical and complex designs, identification of the appropriate level for tests and full reporting of outcomes
☒	☐	Estimates of effect sizes (e.g. Cohen's d, Pearson's r), indicating how they were calculated

Our web collection on statistics for biologists contains articles on many of the points above.

Software and code

Policy information about availability of computer code

Data collection	Real-time fluorescence was determined using the Bio-Rad CFX96 Touch™ Real-Time PCR Detection System. Visual detection was accomplished through imaging the tubes in the LED blue light illuminator or the Bio-Rad ChemiDoc™ MP Imaging System with its built-in UV channel. The endpoint fluorescence was the raw fluorescence determined by the Real-Time PCR Detection System. Gel imaging was conducted using the Bio-Rad ChemiDoc™ MP Imaging System.
Data analysis	GraphPad Software Prism 8 was used to generate real-time fluorescence curves, including the unpaired t-test for the statistical analysis.

For manuscripts utilizing custom algorithms or software that are central to the research but not yet described in published literature, software must be made available to editors and reviewers. We strongly encourage code deposition in a community repository (e.g. GitHub). See the Nature Research guidelines for submitting code & software for further information.

Data

Policy information about availability of data

All manuscripts must include a data availability statement. This statement should provide the following information, where applicable:

- Accession codes, unique identifiers, or web links for publicly available datasets
- A list of figures that have associated raw data
- A description of any restrictions on data availability

The authors declare that the data supporting the findings of this study are available within the article and its supplementary information files or from the corresponding author upon reasonable request. Sequence information used in this study was from National Center for Biotechnology Information (NCBI) with GenBank accessions of MT688716.1 and LC528233.1 (https://www.ncbi.nlm.nih.gov/). Multiple sequence alignments analysis of 4663 SARS-CoV-2 genomes (as of August 14, 2020) are available from GISAID (https://www.gisaid.org/epiflu-applications/next-hcov-19-app/).The source data involving endpoint fluorescence, threshold time to positive, and the number of mutations in Fig. 2b, c, 3a, b, and 5c, and Supplementary Fig. 3b, c, 4b, c, 5b, c, 6a-e, 7c, 8c, 12b, and c, are provided

as a Source Data file

Field-specific reporting

Please select the one below that is the best fit for your research. If you are not sure, read the appropriate sections before making your selection.

- [x] Life sciences
- [ ] Behavioural & social sciences
- [ ] Ecological, evolutionary & environmental sciences

For a reference copy of the document with all sections, see nature.com/documents/nr-reporting-summary-flat.pdf

Life sciences study design

All studies must disclose on these points even when the disclosure is negative.

Sample size	The sample size in this study was not calculated. Since our aim is just to demonstrate the feasibility of the AIOD-CRISPR assay for clinical sample testing, we detected 28 clinical samples (8 COVID-19 positive and 20 negative). Replicate tests (two, three or more repeats) using clinical samples, plasmid templates and synthetic RNA for AIOD-CRISPR assays were run to ensure experimental reproducibility.
Data exclusions	No data exclusions
Replication	At least three replicates were set up for each real-time and endpoint fluorescence detection of plasmid or synthetic RNA templates to demonstrate the findings were reliably reproduced. For real sample testing, two independent experiments were carried out to make sure the detection results were reliable.
Randomization	Plasmid templates were commercialized plasmids inserted with the target sequence which was highly conserved for primer design to detect SARS-CoV-2. Based on the selected target sequence, synthetic RNA was made using the commercialized kit. They have to be non-randomly selected in order to evaluate the detection performance of our established method.
Blinding	Clinical samples were tested by an individual who was blinded to the RT-PCR results.

Reporting for specific materials, systems and methods

We require information from authors about some types of materials, experimental systems and methods used in many studies. Here, indicate whether each material, system or method listed is relevant to your study. If you are not sure if a list item applies to your research, read the appropriate section before selecting a response.

Materials & experimental systems

n/a	Involved in the study	
x		Antibodies
x		Eukaryotic cell lines
x		Palaeontology and archaeology
x		Animals and other organisms
x		Human research participants
x		Clinical data
x		Dual use research of concern

Methods

n/a	Involved in the study	
x		ChIP-seq
x		Flow cytometry
x		MRI-based neuroimaging
